# Anodal Transcranial Direct Current Stimulation of Anterior Cingulate Cortex Modulates Subcortical Brain Regions Resulting in Cognitive Enhancement

**DOI:** 10.3389/fnhum.2020.584136

**Published:** 2020-12-16

**Authors:** Ahsan Khan, Xin Wang, Chun Hang Eden Ti, Chun-Yu Tse, Kai-Yu Tong

**Affiliations:** ^1^Biomedical Engineering Department, The Chinese University of Hong Kong, Hong Kong, China; ^2^Department of Social and Behavioural Science, City University of Hong Kong, Hong Kong, China; ^3^Hong Kong Brain and Mind Institute, The Chinese University of Hong Kong, Hong Kong, China

**Keywords:** transcranial direct current stimulation (tDCS), stroop task, anterior cingulate cortex (ACC), functional magnetic resonance imaging (fMRI), functional connectivity

## Abstract

Transcranial direct current stimulation (tDCS) has been widely utilized in research settings and modulates brain activity. The application of anodal tDCS on the prefrontal cortex has indicated improvement in cognitive functioning. The cingulate cortex, situated in the medial aspect of the prefrontal cortex, has been identified as a core region performing cognitive functions. Most of the previous studies investigating the impact of stimulation on the prefrontal cortex stimulated the dorsolateral prefrontal cortex (DLPFC), however, the impact of stimulation on cingulate has not been explored. The current study investigates the effect of stimulation on the resting-state functional connectivity of the anterior cingulate cortex with other regions of the brain and changes in behavioral results in a color-word Stroop task, which has repeatedly elicited activation in different regions of the cingulate. Twenty subjects were randomly assigned to the experimental and sham group, and their medial prefrontal area was stimulated using MRI compatible tDCS. Resting-state functional magnetic resonance imaging (rs-fMRI) and cognitive Stroop task were monitored before, during, and after the stimulation. Neuroimaging results indicated a significant decrease in resting-state functional connectivity in the experimental group during and after stimulation as compared to before stimulation in two clusters including right insular cortex, right central operculum cortex, right frontal operculum cortex and right planum polare with the left anterior cingulate cortex (L-ACC) selected as the seed. The behavioral results indicated a significant decrease in reaction time (RT) following stimulation in the experimental group compared to the sham group. Moreover, the change in functional connectivity in subcortical regions with L-ACC as the seed and change in RT was positively correlated. The results demonstrated that ACC has a close functional relationship with the subcortical regions, and stimulation of ACC can modulate these connections, which subsequently improves behavioral performance, thus, providing another potential target of stimulation for cognitive enhancement.

**Clinical Trial Registration**: ClinicalTrials.gov Identifier: NCT04318522.

## 1. Introduction

The process of cognitive control is one of the mysteries in cognitive neuroscience, and several studies have been performed to understand how millions of neurons in the prefrontal cortex interact with each other to exhibit a goal-directed behavior (Shackman et al., 2011; Gratton et al., 2018; Wu et al., 2019). Recent evidence suggests that we can manipulate the brain activity through external stimulation and transcranial direct current stimulation (tDCS), a non-invasive brain stimulation method, has emerged as a prime tool for manipulating brain activity (Nitsche et al., 2008). The last decade has seen a sharp increase in the use of stimulation methodologies not just in understanding cognition but also in treating Parkinson's disease (Biagioni et al., 2018), motor rehabilitation following stroke (Bao et al., 2020), chronic pain (O'Connell et al., 2018), and other anxiety disorders (Kuo et al., 2014). Stimulation at the target region provides a causal inference on how the directly induced neural alterations influence behavioral changes, establishing a better understanding of the brain-behavior relationship. In this study, we utilized tDCS to understand the impact of stimulation on the prefrontal region of the brain and investigate if it can interfere with the cognitive control in the human brain.

Previous prefrontal tDCS studies mainly targeting the lateral prefrontal cortex (LPFC) region have reported that stimulation can influence a wide range of cognitive functions ranging from low-level attentional processes to higher-order decision making and working memory functions (Boonstra et al., 2016; Westphal et al., 2019) with some conflicting results (Berryhill et al., 2014; Tremblay et al., 2014). However, the impact of tDCS on the cingulate cortex is not clearly understood. Activation of different regions of cingulate during cognitive control processes (Dum and Strick, 1993; Medford and Critchley, 2010; Rolls, 2019) and a central location of cingulate gives a notion that the stimulation of this region could also provide a better insight into cognitive control processes and possibly enhance cognitive functioning of the human brain.

The cingulate cortex is a complex structure having anatomical connections with various brain regions and has reported involvement in cognitive control functions (Dum and Strick, 1993; Medford and Critchley, 2010; Rolls, 2019). Traditionally, cingulate has been subdivided into three subregions: the anterior cingulate cortex (ACC), the midcingulate cortex (MCC), and the posterior cingulate cortex (PCC) (Tzourio-Mazoyer et al., 2002). ACC has reported activations in certain executive functions, including attention allocation, perception, anticipation, decision making, and impulse control (Paus, 2001). The role of MCC is controversial; however, it has elicited activation in goal-directed behaviors (Tolomeo et al., 2016). PCC, a key component in the default mode network (DMN), has shown heterogeneous connections with widespread brain regions. It plays an active role in cognitive control (Leech et al., 2012) and has shown changes with learning, memory, and task engagement (Pearson et al., 2011).

Stroop Task is one of the most widely used paradigms to study cognitive control and is termed as the “gold standard” of attentional measures (MacLeod, 1992). The most basic color-word version of stroop task involves goal-directed selective attention (West and Alain, 2000), inhibition (Bélanger et al., 2010), conflict detection and conflict resolution (Coderre et al., 2011) along with other cognitive control processes depending on the paradigm. Several brain regions have reported activation during the stroop task including dorsolateral prefrontal cortex (DLPFC) (Vanderhasselt et al., 2009) and different regions of cingulate cortex (Carter and Van Veen, 2007; Badzakova-Trajkov et al., 2009). Specifically, the ACC has been reported to mediate conflict adaptation when conflicting information is simultaneously presented (Kim et al., 2014). As it occurs in incongruent trials of the Stroop task when the color of the word is different than the word itself, and the participant has to resolve the conflict and respond to the color of the word, which takes longer processing time than congruent trials where the word and the color are same (Carter and Van Veen, 2007; Kim et al., 2014).

This study's objective was to understand the impact of tDCS on the ACC and executive functions using the Stroop task. First, functional magnetic resonance imaging (fMRI) was used to explore if stimulation of anterior region of cingulate with a small direct current can affect the resting-state functional connectivity of ACC with other brain regions during the stimulation and immediately after the stimulation. Second, we examined if stimulation can enhance performance in cognitive stroop task and if changes in stroop task have a carryover effect in resting-state connectivity in other cingulate regions including MCC and PCC. Third, we investigated if there is an association between stimulation induced resting-state fMRI changes in ACC with behavioral changes. We hypothesized that stimulation would modulate the ACC connectivity with other brain regions and facilitate conflict adaptation, thus resulting in an improvement in performance in incongruent trials of the stroop task.

## 2. Materials and Methods

**Figure 1:**
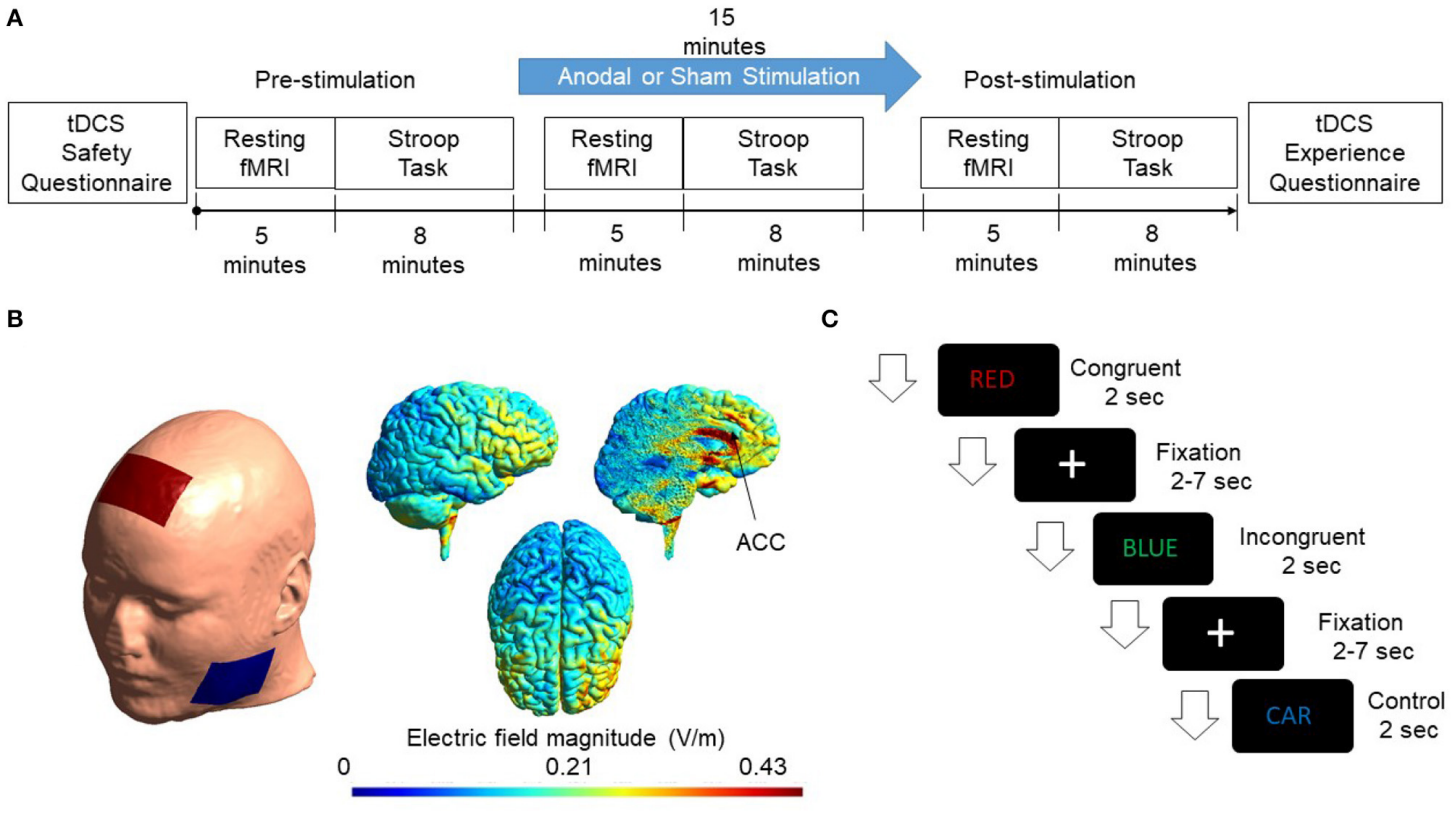
**(A)** Flow chart of the experimental design is shown. Subjects were randomly assigned to the Experimental & Sham group. The cingulate cortex was stimulated for 15 min with a current intensity of 2 mA. Resting-state fMRI and Stroop task were administered pre, during, and post-stimulation. **(B)** A FEM model was used to simulate the electric field distribution adopted in this study. Electrodes were modeled as rubber electrodes with a sponge soaked in saline solution. Anode was placed at Fz according to the International 10/20 system. The cathode was placed at the cheek to minimize the effect of cathodal stimulation. The applied current was 2mA. Simulated electric field distribution in sagittal, axial, and the sliced sagittal view is shown. **(C)** The figure shows the presentation of events. Congruent, incongruent, and control trials appeared randomly on the screen. Each event occurred for 2 s while a fixation cross appeared after each trial with a jitter of 2–7 s.

The current study adopted a single-blinded, placebo-controlled, randomized parallel-group design. Volunteers were screened for inclusion and exclusion criteria (see below) before enrollment in the study. They were instructed about the task on the day of the experiment, and written consent was taken from all the participants. Subjects were randomly assigned to the experimental and sham groups with ten subjects in each group. At pre-stimulation, resting-state fMRI was collected for 5 min before the participants performed a color-word version of the Stroop task, which lasted for 8 min. Following pre-stimulation measurements, tDCS was performed. During the 15-min stimulation, a 5-min resting-state fMRI was acquired at the beginning of the stimulation, followed by a Stroop task. Following stimulation, resting-state fMRI was collected, and the Stroop task was performed. For the sham group, the stimulation ramped up to 2 mA for 30 s and then ramped down in the next 30 s to 0 mA. No stimulation followed this for the next 15 min. The study design is shown in Figure 1A.

### 2.1. Subjects

Twenty healthy subjects (15 males and five females) were recruited for this study with a mean age of 23.61±2.77 years. However, only 18 subjects (13 males and five females) were able to finish the experiment, of which 10 were in the experimental group and eight in the sham stimulation group. Two subjects were not comfortable with the fMRI procedures and not able to finish the study. The inclusion criteria were: Right handedness and Age ≥ 18, normal or corrected-to-normal vision. All the participants were undergraduate or post-graduate students at The Chinese University of Hong Kong. The exclusion criteria were: a history of any neurological disease, any psychological or psychiatric disease, significant visual or auditory impairment, tDCS, or fMRI contraindications including implanted metallic or electronic devices, seizures, or convulsions, and pregnancy. The study was conducted in accordance with the Helsinki declaration ethical standards and approved by the Joint Chinese University of Hong Kong New Territories East Cluster Clinical Research Ethics Committee.

### 2.2. Transcranial Direct Current Stimulation

#### 2.2.1. Stimulation Parameters

A direct current of 2 mA was administered for 15 min using conventional transcranial direct current stimulator (tDCS) (DC-Stimulator, Neuroconn GmbH, Ilmenau, Germany). Two conductive sponges act as cathode and anode with current flowing from one sponge to the other. The anode was placed at Fz location based on the 10–20 system as done in previous studies (To et al., 2018) while cathode was placed at the cheek. Conductive saline solution was applied on the sponge electrode to keep the impedance below 15 kΩ.

tDCS is bipolar, i.e., both cathode and anode impact the brain in opposite directions. Anodal stimulation increases the activation of brain activity while cathodal results in a decrease in brain activity (Stagg and Nitsche, 2011). Most of the studies reported an increase in brain excitability as a result of anodal stimulation. Thus, we utilized anodal stimulation in the present study. While performing anodal stimulation, we want to minimize the impact of cathodal stimulation. One possible methodology is to place anode over the target region in one hemisphere and cathode over the same region in the other hemisphere (Kadosh et al., 2010). However, this placement results in activation of one hemisphere and deactivation of the other, and any changes in behavioral outcome could result from either of them, which makes it difficult to conclude any specific function of the target brain area. Another possible placement methodology is to place the anode over the target area and cathode on one of the cheeks (Tseng et al., 2012) to minimize the impact of the cathode as much as possible. The field produced by cathodal stimulation only affects facial muscles and gums, which provide the least resistance path to the current, thus reducing the cathode's impact on the brain. Scalp-cheek placement methodology was used in the study.

Furthermore, the human skull offers resistance to the current flow, and a considerable amount of current is shunted by the skull. Approximately, 75% of the current is attenuated by the scalp tissues and the skull (Vöröslakos et al., 2018). The duration of the current also plays an essential rule in determining the impact of stimulation. A meta-analysis examining the impact of stimulation reported that stimulation duration of more than 10 min and a current density of more than 0.029 mA/cm^2^ could have a significant impact on behavioral results (Hill et al., 2016). Thus, we chose 2mA current with a duration of 15 min.

#### 2.2.2. Simulation of the Electric Field Generated by tDCS

A Finite element model (FEM) was generated to simulate the electric field and current density distribution induced from tDCS using SIMNIBS 3.0.1 (Thielscher et al., 2015). FEM head mesh was first generated using T1 structural MRI data provided by the software. The head mesh was segmented as a six-compartment model with segmented scalp, skull, gray matter, white matter, cerebrospinal fluid (CSF), and eyes. Simulations were then initialized by placing modeled electrode montage on the surface of the scalp. Electrodes were configured as 5 × 7 cm rubber electrodes and placed in a customized location to mimic the actual stimulation montage. Electric field and current density distributions were calculated using volume normalized anisotropic conductivities (Güllmar et al., 2010). The diffusion tensor data provided by the software was normalized to have the same trace and rescaled in accordance with the reference isotropic conductivity of respective tissues (Opitz et al., 2015). Simulated results are shown in Figure 1B.

### 2.3. Color-Word Stroop Task

Stroop task is considered a gold standard for studying executive functions of the human brain. In the standard color-word version of the Stroop task, a word is presented in different colors. The color could be the word itself, for example, the word “RED” written in red color (congruent trials), or it could be different from the word, for example, the word “BLUE” written in green color (incongruent trials) (Zysset et al., 2001) or it could be a neutral trial in different colors in which the word used is not a color, for example, the word “CAR” written in blue color (control trials). For congruent and incongruent trials, there were four color words, and each of them was paired with each of the four colors. For the control trials, ten neutral words, which were not the name of a color or with a dominant color, were paired with the four colors to produce the neutral/control word stimuli. The experiment was presented using E-prime 3.0 (Schneider et al., 2013). Subjects were asked to respond to the color of the word and were given time to memorize the combination prior to the experiment. Two two-button panels were used inside the scanner numbering from 1 to 4. Subjects were asked to press 1 for blue, 2 for red, 3 for green, and 4 for yellow. One hundred twenty words were presented randomly with 40 words for each condition, i.e., congruent, incongruent, and control. Each of the word events lasted for 2 s with a fixation cross (2–7 s) between trials and an equal probability of occurring. The design of the stimuli presentation is shown in Figure 1C.

### 2.4. Functional Magnetic Resonance Imaging

Functional magnetic resonance imaging (fMRI) has been frequently utilized to understand the functioning of the human brain and the impact of non-invasive stimulation. Functional connectivity analysis is one of the principal methodologies to evaluate the connectivity between two or more brain regions based on their simultaneous engagement in oscillatory activity (Van Den Heuvel and Pol, 2010).

#### 2.4.1. Data Acquisition

Subjects were scanned using a 3T Philips MR scanner with an 8-channel head coil. High resolution T1-weighted anatomical images (TR/TE = 7.47/3.45 ms, flip angle = 8°, 308 slices, voxel size = 0.6 × 1.042 × 1.042 mm^3^) using a T1-TFE sequence (ultrafast spoiled gradient echo pulse sequence) and BOLD fMRI images (TR/TE = 2,000/30 ms, flip angle = 70°, 37 slices/volume, voxel size = 2.8 × 2.8 × 3.5 mm^3^) using a gradient-echo-EPI sequence (gradient-echo echo-planar-imaging sequence) were acquired. fMRI run lasting for 5 min was acquired both before and after the stimulation. During the 15-min stimulation, a 5-min resting-state fMRI run was acquired at the beginning of the stimulation. The subjects were asked to rest while focusing on the white crosshair displayed on the screen and keep still.

#### 2.4.2. Pre-processing

The preprocessing was performed with the default pipeline using the CONN toolbox (Whitfield-Gabrieli and Nieto-Castanon, 2012). Preprocessing steps included slice timing correction, outlier identification from the global signal and framewise displacement, motion correction, and co-registration of the anatomical image to the mean functional volume. All the functional images were then normalized to standard MNI space and resampled to 2 mm. Spatial smoothing was performed with an 8 mm isotropic FWHM Gaussian kernel to increase the signal-to-noise ratio. The resulting images were further processed in additional steps, including band-pass filtering (0.01–0.1 Hz) and regression of motion parameters, signals from white matter, and cerebrospinal fluid.

#### 2.4.3. Functional Connectivity Analysis

**Figure 2:**
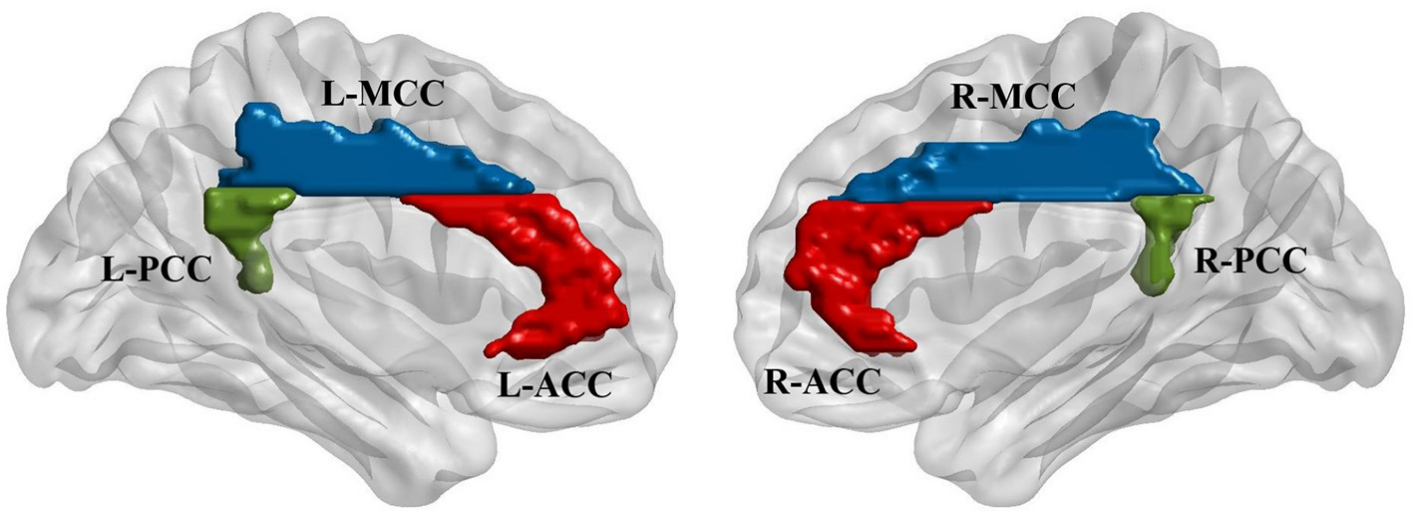
Regions of interest (ROIs) were defined based on automatic anatomic labeling (AAL) atlas. Six cingulate ROIs were defined in both hemispheres including bilateral anterior cingulate cortex (L-ACC & R-ACC), bilateral midcingulate cortex (L-MCC & R-MCC), and bilateral posterior cingulate cortex (L-PCC & R-PCC) as shown in the left and right figures, respectively.

Six regions of interest (ROIs) in the cingulate cortex were selected from the AAL template (Tzourio-Mazoyer et al., 2002; Rolls et al., 2015), including the bilateral anterior cingulate cortex (L-ACC and R-ACC), middle cingulate cortex (L-MCC and R-MCC) and posterior cingulate cortex (L-PCC and R-PCC). The purpose of including bilateral seeds was to investigate if stimulation modulates the interhemispheric connectivity between cingulate seeds. Furthermore, some previous studies have reported significant connectivity differences between left and right cingulate regions (Margulies et al., 2007; Yan et al., 2009). Functional connectivity was calculated between the mean time series of each pair of the ROIs. Besides, for each ROI, a seed-based analysis was performed to explore the functional connectivity between the ROI and the whole brain voxels. Pearson correlation coefficient was used for all the connectivity analysis following by the Fisher r-to-z transformation. The locations of the ROIs generated using BrainNet Viewer (Xia et al., 2013) are shown in Figure 2.

## 3. Statistical Analysis

### 3.1. Behavioral Data

Statistical analyses were conducted with the SPSS 19 statistical software package. (IBM SPSS Statistics, NY, US). At pre-stimulation, a two-sample *t*-test was performed to check for any differences between the groups. For the change in RT in the Stroop task, a repeated measures ANOVA (stim × time × condition) was applied with group (experimental vs. sham) as the between-subjects variable and time (pre vs. during vs. post) and condition (congruent vs. incongruent vs.control) as within-subjects variables. To further study the conditional effects, separate ANOVAs were performed for individual conditions. If a significant ANOVA difference was observed, then *post-hoc* analyses were performed using *t*-tests. For accuracy, ANOVA was performed with group (experimental vs. sham) as between-subject variable and time (pre vs. during vs. post) as a within-subject variable. Bonferroni correction was used for multiple comparison correction. Effect sizes for *t*-tests and ANOVAs were estimated using Cohen's d and partial eta square, respectively (Lakens, 2013).

### 3.2. Neuroimaging Data

CONN 2nd level analysis module was used to perform statistical analysis on the group level data. At pre-stimulation, ROI-voxel and ROI-ROI group differences (experimental vs. sham) were assessed using a two sample *t*-test. To investigate stimulation-induced ROI-voxel and ROI-ROI changes, a 2 × 3 ANOVA was performed for each ROI with the group (experimental vs. sham) as between-subject variable and time (pre vs. during vs. post) as a within-subject variable. If a significant effect was observed for any of the ROI, then one-way ANOVA was performed for each group with three time-periods (pre vs. during vs. post). For ROI-voxel analysis, if significant clusters were identified, then the whole cluster was used as a mask for *post-hoc* analysis, and one way-ANOVA was performed for each group with three time periods (pre vs. during vs. post). For multiple comparison correction, random field theory parametric statistics were utilized. First, a statistical parametric map was estimated with a voxel threshold of *p* < 0.001, and a series of non-overlapping clusters were identified using the 18-connectivity criterion on neighboring voxels. Then, false discovery rate (FDR) corrections (Chumbley et al., 2010) with a cluster threshold of *p* < 0.05 were applied. Further, Bonferroni correction was applied on two anterior cingulate seeds and four mid and posterior cingulate seeds.

### 3.3. Brain-Behavior Correlations

To further understand the relationship between resting-state fMRI changes and behavioral changes, Spearman's correlation analysis was performed between significant changes in RT and significant changes in functional connectivity for the experimental group. Bonferroni correction was used for multiple comparison correction.

## 4. Results

### 4.1. Demographic and Descriptive Information

A comparison between the ages of the participants in the experimental and sham group did not show any significant difference (*t* = 0.148, *p* = 0.884). Subjects in the experimental group reported mild to moderate itchiness on the skin and scalp following stimulation. No other tDCS related experiences were reported by any of the subjects.

### 4.2. Behavioral Results

ANOVA results at the pre-stimulation revealed a significant effect between conditions (*F* = 5.892, *p* = 0.007, $\eta_{p}^{2}=0.269$). Further analysis showed that the subjects were slower for incongruent trials (Mean = 882.781 ± 148.411 s) as compared to congruent (Mean = 757.475 ± 106.851 s) and control trials (Mean =796.062 ± 122.045 s). Comparison between the RT of experimental and sham group at pre-stimulation did not show any significant difference for the congruent (*t* = 0.163, *p* = 0.873), incongruent (*t* = 1.397, *p* = 0.182) and control (*t* = 1.543, *p* = 0.142) trials.

**Figure 3:**
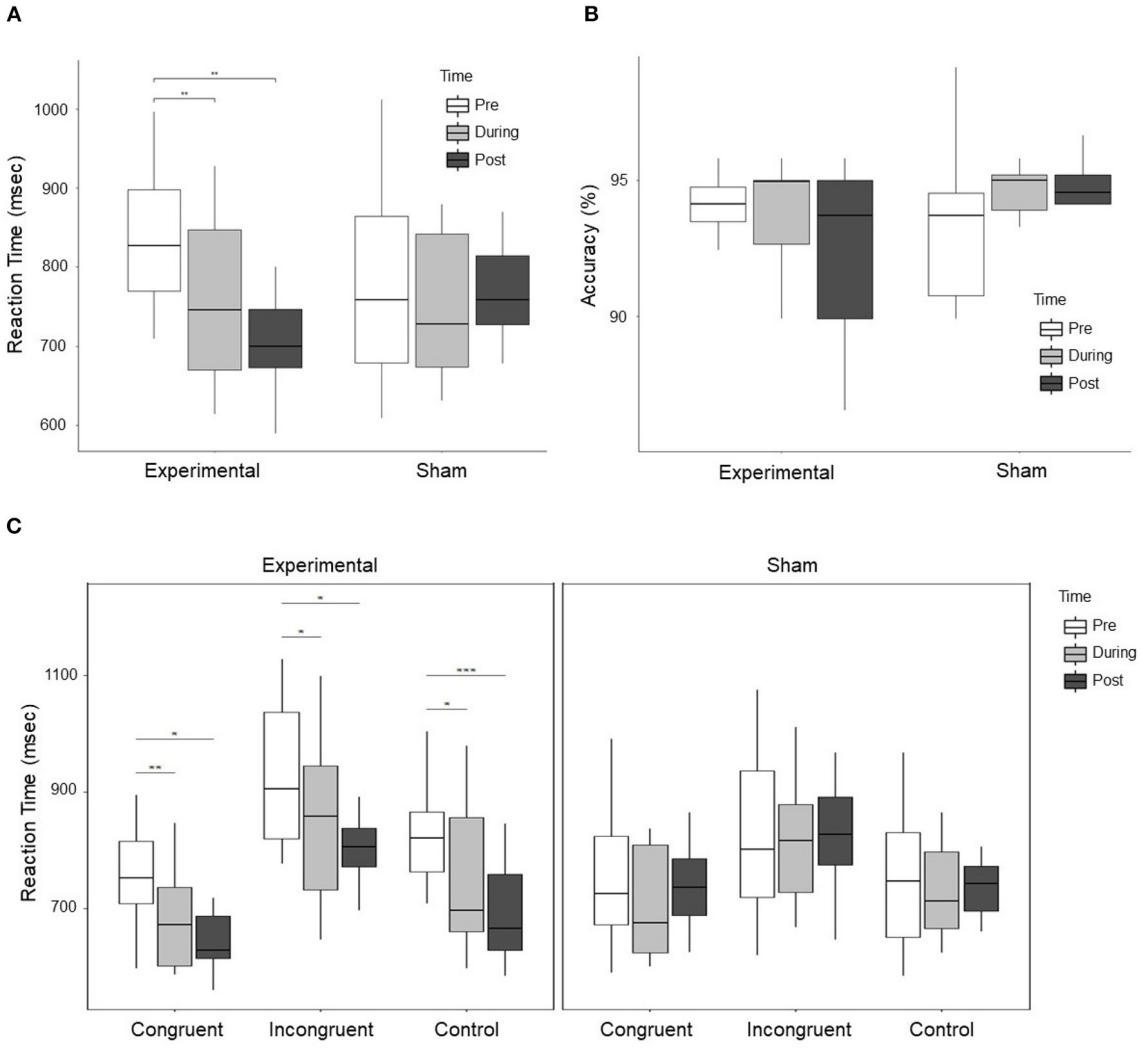
**(A)** Shows the RT of subjects in the experimental and sham group for three time periods, i.e., pre, during, and post-stimulation. A significant Time × Group effect between experimental and sham group was observed. Further exploration indicated a significant decrease in RT in the experimental group during stimulation as compared to pre-stimulation, and post-stimulation as compared to pre-stimulation. **(B)** Shows the accuracy of subjects response to cognitive stroop task. No significant effect was observed for the change in accuracy, however, the trend shows that the accuracy reduced in the experimental group and improved in the sham group. **(C)** Shows the change in RT in the experimental and sham group for each condition, i.e., congruent, incongruent, and control trials. The left figure displays the change in RT for all the conditions in the experimental group, while the right figure shows the change in RT for all the conditions in the sham group. In the experimental group, a significant decrease in RT for congruent, incongruent, and control trials was observed during stimulation and post-stimulation as compared to pre-stimulation. For control trials, a significant decrease in RT was observed post-stimulation as compared to during stimulation. In the figure * represents *p* < 0.05, ** represents *p* < 0.01, and *** represents *p* < 0.001.

Repeated measures ANOVA results with stimulation group (experimental vs. sham) as between-subject variable and time (pre. vs. during. vs. post) and condition (congruent vs. incongruent vs. control) as within-subject variable showed a significant difference for Time (*F* = 8.312, *p* = 0.001, $\eta_{p}^{2}=0.342$), Condition (*F* = 66.204, *p* < 0.001, $\eta_{p}^{2}=0.805$), Time × Group (*F* = 5.494, *p* = 0.009, $\eta_{p}^{2}=0.256$), and Group × Condition (5.659, *p* = 0.008, $\eta_{p}^{2}=0.261$). However, no significant Time × Group × Condition interaction was observed (*F* = 0.457, *p* = 0.767, $\eta_{p}^{2}=0.028$). Further ANOVA was performed for experimental and sham group separately with time (pre vs. during vs. post) as a within-subject variable. A significant effect was observed (*F* = 11.176, *p* < 0.001, $\eta_{p}^{2}=0.554$) for the experimental group but no significant difference (*F* = 0.543, *p* = 0.593, $\eta_{p}^{2}=0.072$) was observed for the sham group. Paired *t*-test were conducted in experimental group between time periods (pre vs. during, pre vs. post, and during vs. post). A significant decrease in RT was observed during stimulation (*t* = 4.363, *p* = 0.002, *d* = 0.745), and post-stimulation (*t* = 4.850, *p* = 0.001, *d* = 1.253) as compared to pre-stimulation. The change in RT in the experimental and sham group are shown in Figure 3A. Furthermore, no significant effect was observed for accuracy (*F* = 1.214, *p* = 0.310, $\eta_{p}^{2}=0.044$) with group (experimental vs. sham) as a between-subject variable and time (pre vs. during vs. post) as a within-subject variable. At pre-stimulation experimental group showed an accuracy of (*Mean* = 93.446 ± 2.907%) and sham group showed an accuracy of (*Mean* = 93.49 ± 3.07%). The accuracy changes are shown in Figure 3B.

ANOVA results for individual conditions showed a significant group × time effect for congruent trials (*F* = 3.603, *p* = 0.039, $\eta_{p}^{2}=0.184$), marginally significant for incongruent trials (*F* = 3.278, *p* = 0.051, $\eta_{p}^{2}=0.170$) and strongly significant for control trials (*F* = 6.284, *p* = 0.005, $\eta_{p}^{2}=0.282$). For *post-hoc* analysis, we conducted paired *t*-test in the experimental and sham group for congruent, incongruent, and control trials between time periods (pre vs. post, pre vs. during, and during vs. post). For congruent trials in the experimental group, a significant decrease in RT was observed during stimulation (*t* = 4.711, *p* = 0.001, *d* = 0.888) and post-stimulation (*t* = 3.749, *p* = 0.005, *d* = 1.176) as compared to pre-stimulation. For incongruent trials in the experimental group, a significant decrease was observed during stimulation (*t* = 2.949, *p* = 0.016, *d* = 0.552) and post-stimulation (*t* = 3.681, *p* = 0.005, *d* = 0.930) as compared to pre-stimulation. For control trials in the experimental group, a significant decrease was observed during stimulation (*t* = 3.335, *p* = 0.009, *d* = 0.757), and post-stimulation (*t* = 6.406, *p* < 0.001, *d* = 1.5451) as compared to pre-stimulation and a significant decrease in post-stimulation (*t* = 2.695, *p* = 0.025, *d* = 0.520) as compared to during stimulation. Changes in RT in the experimental and sham group for individual conditions are shown in Figure 3C. No significant effect was observed for individual conditions in the sham group.

### 4.3. Functional Connectivity Analysis

#### 4.3.1. ROI to Voxels

**Figure 4:**
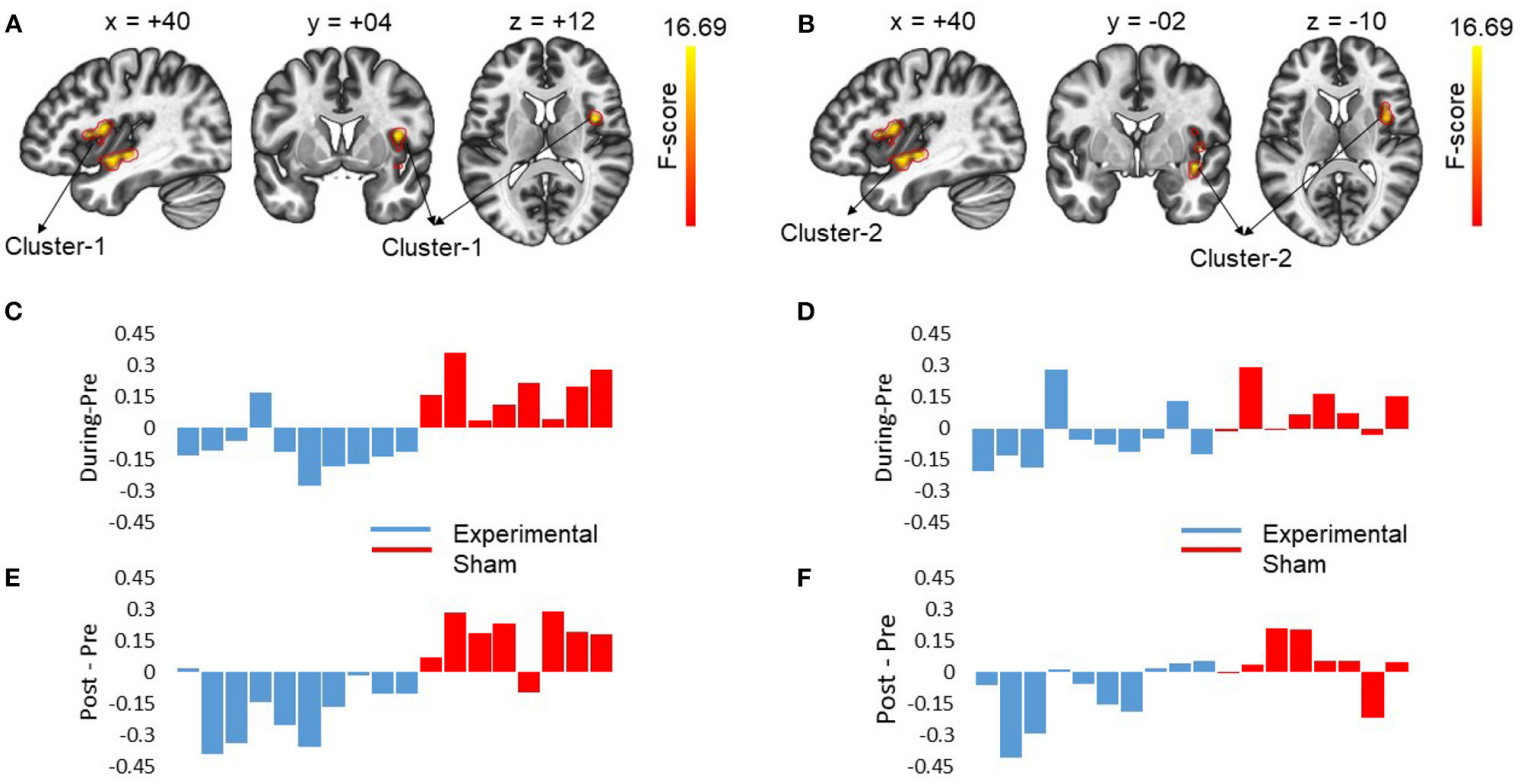
The figures show the changes in functional connectivity for cluster 1 [+40 +04 +12] and cluster 2 [40 −02 −10] with L-ACC selected as the seed. The results are obtained by calculating ANOVA difference between groups (experimental vs. sham) as between-subject variable and time (pre vs. during vs. post) as a within-subject variable. **(A)** Sagittal, coronal and axial slices of brain from left to right with significant changes in connectivity between L-ACC and cluster 1 covering right anterior insular cortex (R-AIC) and right planum Polare are shown. The center of cluster 1 [+40 +04 +12] was chosen as the target slice. **(B)** shows the sagittal, coronal, and axial slices of brain from left to right with significant changes in connectivity between L-ACC and cluster 2 covering right central opercular cortex, right posterior insular cortex, and right frontal opercular cortex. The center of cluster 2 [+40 −02 −10] was chosen as the target slice. **(C)** Displays the difference between during stimulation connectivity and pre-stimulation connectivity for cluster 1. **(D)** Depicts the difference between during stimulation connectivity and pre-stimulation connectivity for cluster 2. **(E)** Shows the difference between post-stimulation connectivity and pre-stimulation connectivity for cluster 1. **(F)** Shows the difference between post-stimulation connectivity and pre-stimulation connectivity for cluster 2. The results indicate that stimulation resulted in a decrease in connectivity in the experimental group which lasted beyond stimulation period. However, for the sham group an opposite effect was observed. Moreover, the impact of stimulation showed inter-subject variability.

At pre-stimulation, no significant difference was observed between the experimental and sham groups. The ANOVA results with group (experimental vs. sham) as between-subject variable and time (pre vs. during vs. post) as a within-subject variable showed a significant difference in connectivity for two clusters including R anterior insular cortex, Right Planum Polare (*F* = 16.69, *p*-corrected = 0.010685, $\eta_{p}^{2}=0.481$) and R Central Opercular Cortex, R Frontal Operculum Cortex (*F* = 16.69, -corrected = 0.011217, $\eta_{p}^{2}=0.481$) with L-ACC as the seed as shown in Table 1. Axial, sagittal, and coronal views are shown in Figures 4A,B at the center of cluster 1 and cluster 2, respectively. Further, one-way ANOVA was performed for experimental and sham groups separately with time (pre vs. during. vs. post) as a within subject variable. For the experimental group, we observed a significant difference (*F* = 10.407, *p* = 0.001, $\eta_{p}^{2}=0.536$) between time periods (pre vs. during vs. post). For the sham group, we also observed a significant difference (*F* = 6.571, *p* = 0.010, $\eta_{p}^{2}=0.484$) between time periods (pre vs. during vs. post). For *post-hoc* analysis we performed paired *t*-tests which showed a significant decrease in connectivity during stimulation (*t* = 4.566, *p* = 0.001, *d* = 1.253), and post-stimulation (*t* = 2.490, *p* = 0.034, *d* = 0.789) as compared to pre-stimulation in the experimental group and a significant increase in connectivity during stimulation (*t* = 3.771, *p* = 0.007, *d* = 1.388) as compared to pre-stimulation in the sham group. No significance was observed for post-stimulation (*t* = 1.697, *p* = 0.133, *d* = 0.630) as compared to pre-stimulation in the sham group.

**Table 1:** The table shows 2 × 3 ANOVA results with group (experimental vs. sham) as a between-subject variable and time (pre vs. during vs. post) as a within subject variable with L-ACC selected as the seed.

**Clusters**	**Cluster (x, y, z)**	**Cluster size**	***p*-FDR**	**Regions**
Cluster 1	[+40 +04 +12]	145 voxels	0.010685	R Ant Insular Cortex
				R Planum Polare
Cluster 2	[+40 −02 −10]	170 voxels	0.011217	R Central Opercular Cortex
				R Pos Insular Cortex
				R Frontal Operculum Cortex

*p-FDR = p-value after false discovery rate correction, R = right, Pos = posterior, Ant = anterior*.

#### 4.3.2. ROI-ROI

ANOVA results did not show any significance for ROI—ROI functional connectivity between any of the defined seeds.

### 4.4. Brain-Behavior Relationship

**Figure 5:**
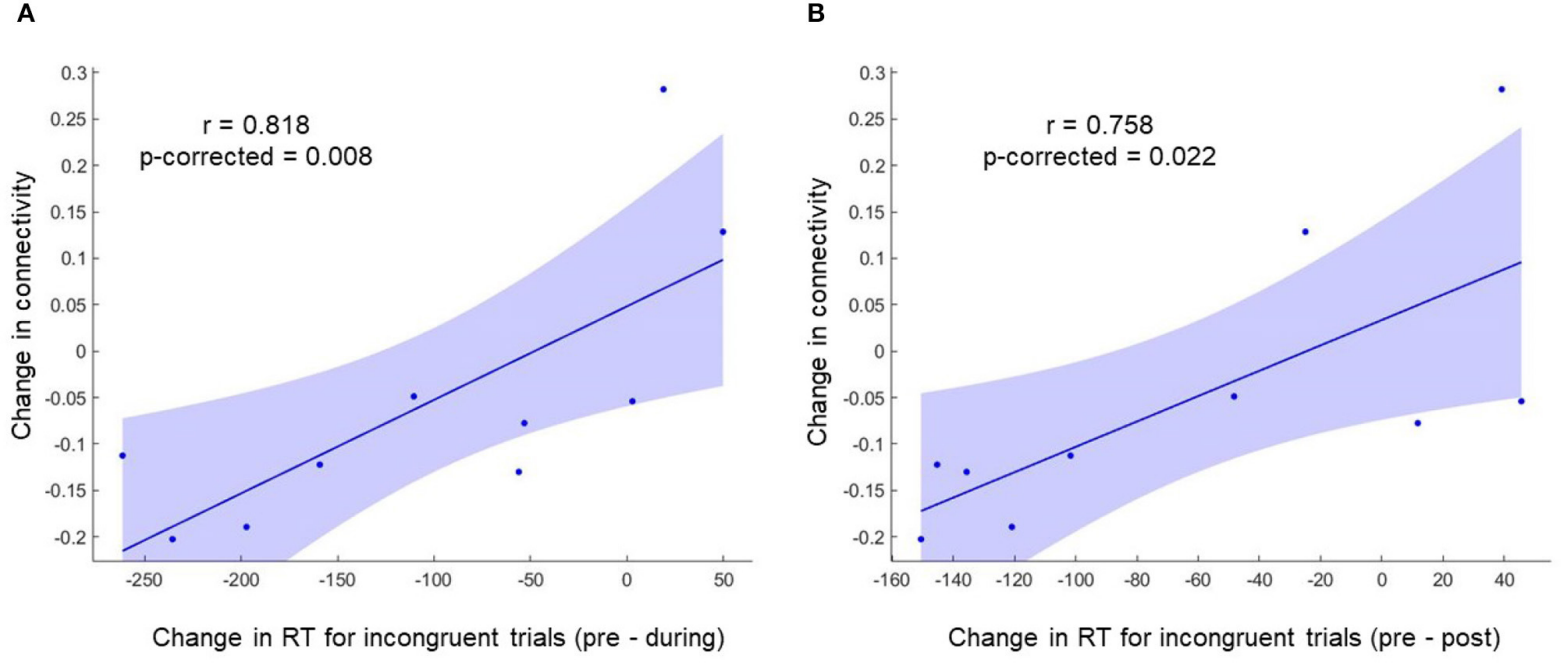
A significant positive correlation was observed between change in RT for incongruent trials and change in functional connectivity for cluster 1. **(A)** Shows the correlation between change in RT (During—Pre) for incongruent trials and change in functional connectivity for cluster 1 (During—Pre). **(B)** Shows the correlation between change in RT (Post—Pre) for incongruent trials and change in functional connectivity for cluster 1 (During—Pre).

To investigate the relation between neuroimaging results and behavioral results, we performed a Spearman's rho correlation between the change in RT for all the conditions and the change in functional connectivity for L-ACC with significant clusters. Two 5mm ROIs were defined at the center of cluster 1 [+40 +04 +12] and cluster 2 [+40 −02 −10] in MNI space. The connectivity changes during stimulation for cluster 1 and cluster 2 are shown in Figures 4C,D, respectively. Similarly, post-stimulation connectivity variations for cluster 1 and cluster 2 are shown in Figures 4E,F, respectively. A correlation analysis was performed on the experimental group to assess stimulation related brain-behavior relationships. A significant positive correlation (*r* = 0.818, *p*-corrected = 0.008) was observed between decrease in RT (Pre—During) for incongruent trials and decrease in connectivity (pre—during) for cluster 1 as shown in Figure 5A and a positive correlation (*r* = 0.758, *p*-corrected = 0.022) between decrease in RT (pre—post) for incongruent trials and decrease in connectivity (pre—during) for cluster 1 as shown in Figure 5B.

## 5. Discussion

Distinct neural mechanisms have been reported to mediate various aspects of cognitive control (Gratton et al., 2018). However, these mechanisms are not clearly understood due to the involvement of various brain regions in cognitive processing. Brain stimulation offers a possible approach to understand these mechanisms by modulating specific regions with small electric currents and observe changes in the brain signals and behavioral outcome. Here in this study, we aimed to stimulate the cingulate cortex using tDCS to observe the changes in the resting-state brain activity and the subsequent changes in the performance during a cognitive Stroop task.

### 5.1. Behavioral Results

In the behavioral results, we observed a significantly faster RT in the experimental group as compared to the sham group. This improvement in RT was observed during and after the stimulation for congruent, incongruent, and control trials in the experimental group with no significant change in accuracy. Such behavioral gains have been observed in other stimulation related studies targeting different regions of the frontal cortex (Martin et al., 2014; Gbadeyan et al., 2016).

However, the results contradict a recent study that involved stimulation of ACC using high-definition tDCS (HD-tDCS) (To et al., 2018). They reported decreased RT in the incongruent trials of cognitive Stroop task after anodal stimulation, while no effect was observed for congruent and control trials. This can be related to the fact that HD-tDCS is more focal than conventional tDCS, and specific brain regions can be targeted using HD-tDCS (Villamar et al., 2013). However, conventional tDCS impacts broad areas in cortical and subcortical regions resulting in a more general improvement in all the trials irrespective of the trial type. Furthermore, an information theory account of cognitive control suggests that the role of ACC is not just conflict detection, as suggested by several other studies (Kerns et al., 2004; Carter and Van Veen, 2007); rather, it has a more general role in cognitive control (Fan, 2014). According to this theory, ACC, Anterior Insular cortex (AIC), and other brain areas are information processing entities that process information, and conflict is a special case of this information processing. Thus, stimulation of ACC could result in a general improvement in behavioral performance, as indicated in the current study.

### 5.2. Neuroimaging Results

Studies have reported that the functional organization of various networks exist in the brain even when the participant is not subjected to any external attentional demands, i.e., during rest (Fox et al., 2006). Our study investigated stimulation-induced resting-state connectivity changes in different regions of cingulate. A significant decrease in the functional connectivity between the L-ACC and R-Insula, along with other subcortical regions, was observed as a result of stimulation in the experimental group. Further analysis revealed two clusters of change, one including the anterior insular cortex (AIC) and one including posterior insular cortex (PIC). The insular cortex has been reported to play a critical role in attentional processes and cognitive control (Seeley et al., 2007; Menon and Uddin, 2010). Moreover, the ACC and the AIC are two significant parts of the cognitive control network and have shown a close functional relationship across emotional processing, memory, cognition, sensation, and other behavioral contexts (Medford and Critchley, 2010; Menon and Uddin, 2010). Furthermore, Right AIC has been implicated to play a critical role in switching between two other major networks the default mode network (DMN) and the executive control network (ECN) during cognitive information processing and has been termed as the bottleneck of cognitive control (Wu et al., 2019).

Similar results have been reported by another study targeting DLPFC, which resulted in a change in the activation in the ACC and a decrease in connectivity between ACC and the whole brain along with subcortical regions (Weber et al., 2014). However, in the mentioned study, stimulation was performed outside the scanner, and they did not observe changes in brain connectivity during the stimulation. Moreover, they used a bilateral electrode montage in which anode was placed above the right DLPFC and cathode over its left counterpart, which makes it difficult to properly interpret the results (Reinhart et al., 2017). A recent study on normal aging indicated that older adults have a stronger connection between ACC and AIC as compared with younger adults, which was attributed to cognitive decline in elderly subjects (Cao et al., 2014). Furthermore, meditation studies have reported that the meditation modulates the activity between left ACC and insula thus creating interhemispheric frontal asymmetry (Tang et al., 2015). The frontal asymmetry has been reported to be an index for positive mood (Tang et al., 2015) which may enhance cognitive performance. These studies suggest that altered resting-state connectivity between L-ACC and insular regions, as shown in the present study might indicate enhanced cognitive functioning.

Moreover, an increase in the connectivity for the sham group following a Stroop task indicates a relaxation phenomenon in relevant brain regions after engaging in a cognitively demanding task. This increase in connectivity following a task has been previously observed in prefrontal regions following a language task (Waites et al., 2005), prefrontal regions in visual cognitive tasks utilizing different faces and complex scenes (Stevens et al., 2010), and bilateral motor regions following a button press task (Tung et al., 2013). However, we could not observe such carryover effect neither in experimental or sham group in other cingulate regions including MCC and PCC which have reported activations during stroop task.

Moreover, we observed inter-individual variability in stimulation induced connectivity changes. This variability in the impact of tDCS has been reported at an individual level in several other studies (Dedoncker et al., 2016; Lefebvre and Liew, 2017). One major reason causing such variations is the difference in individual anatomy, which suggests that the same stimulation protocol applied to different subjects can result in varying electric field strength around the target due to different scalp and skull thickness and white matter anisotropy for each individual (Laakso et al., 2019; Mikkonen et al., 2020). Simulation of electric fields could potentially help to reduce the heterogeneity of the results by determining the optimal stimulation protocols for each individual before comparing the effect of stimulation. Furthermore, in our study, the ROIs were selected based on the AAL template (Tzourio-Mazoyer et al., 2002; Rolls et al., 2015), which parcellates the cingulate into fairly large anterior, mid, and posterior cingulate regions. A recent study proposed a cingulate parcellation scheme at the subregional level, which showed consistency of functional connectivity with anatomical subregions (Jin et al., 2018). Future stimulation-related studies may focus on small cingulate subregions, thus enhancing our understanding of stimulation's impact on cingulate subregional levels. Moreover, utilization of network neuroscience approach (Bassett and Sporns, 2017) in different brain networks, i.e., neurons, circuits, systems, whole brain, behavior, and across various spatial and temporal scales could pave the way for a better understanding of the impact of tDCS on the human brain.

### 5.3. Brain-Behavior Relationship

A correlation analysis was performed between change in RT and change in connectivity between L-ACC and ROIs defined at the center of two clusters. A positive correlation was observed between change in functional connectivity (during stimulation—pre-stimulation) and change in RT for incongruent trials during and post-stimulation as compared to pre stimulation, which suggests that the impact of stimulation on the brain during-stimulation can indicate subsequent improvement in behavioral tasks after stimulation. Previous studies have also reported that the changes in brain activity as a result of stimulation correspond to the changes in behavioral results. For example, anodal stimulation of the medial-frontal region increased learning rates in simple stimulus-response mapping tasks while cathodal stimulation resulted in a decrease in learning (Reinhart and Woodman, 2014). Furthermore, anodal stimulation over the parietal cortex improved acuity and amplitude of event-related potentials (ERPs), while cathodal stimulation resulted in a decrease of both (Reinhart et al., 2016). Here, we demonstrated that tDCS targeted at the ACC could induce neurological changes at resting-state, which further correlated with the changes in the reaction time for incongruent trails. However, such correlations were not observed for congruent and control trials which indicate that stimulation may have impacted relevant brain regions in prefrontal cortex other than ACC resulting in an improvement in these trials which can be further explored in future studies.

### 5.4. Conclusion

We conclude that a 15 min direct current stimulation of 2 mA can penetrate deeper brain structures like cingulate and alters its activity, which results in the reduction of the resting-state functional connectivity of ACC with subcortical regions during stimulation and after stimulation. This decrease in connectivity indicates an enhancement in cognitive functioning, which results in an improvement in performance during the stroop task. Thus, ACC could be another potential target of stimulation to enhance cognitive functioning.

## Data Availability Statement

The raw data supporting the conclusions of this article will be made available by the authors, without undue reservation.

## Ethics Statement

The studies involving human participants were reviewed and approved by Joint Chinese University of Hong Kong New Territories East Cluster Clinical Research Ethics Committee. The patients/participants provided their written informed consent to participate in this study.

## Author Contributions

AK conducted the experiments and analyzed the data with support from XW. CT planned and carried out the simulations. K-YT and C-YT supervised the project. All authors provided critical feedback in analyzing the data and drafting the manuscript.

## Conflict of Interest

The authors declare that the research was conducted in the absence of any commercial or financial relationships that could be construed as a potential conflict of interest.
